# Characterization of *Streptococcus pluranimalium* from a cattle with mastitis by whole genome sequencing and functional validation

**DOI:** 10.1186/s12866-018-1327-0

**Published:** 2018-11-12

**Authors:** Yushan Pan, Haoran An, Tong Fu, Shiyu Zhao, Chengwang Zhang, Genhui Xiao, Jingren Zhang, Xinfang Zhao, Gongzheng Hu

**Affiliations:** 1 College of Animal Science and Veterinary Medicine, Henan Agricultural University, Zhengzhou, China; 2 Center for Infectious Disease Research, School of Medicine, Tsinghua University, Beijing, China; 3 Tsinghua-Peking Joint Center for Life Science, School of Medicine, Tsinghua University, Beijing, China

**Keywords:** *Streptococcus pluranimalium*, Mastitis, Phylogenetic group, Prophage, *mef*(A), *lnu*(C), Carbon catabolism

## Abstract

**Background:**

*Streptococcus pluranimalium* is a new member of the *Streptococcus* genus isolated from multiple different animal hosts. It has been identified as a pathogen associated with subclinical mastitis, valvular endocarditis and septicaemia in animals. Moreover, this bacterium has emerged as a new pathogen for human infective endocarditis and brain abscess. However, the patho-biological properties of *S. pluranimalium* remain virtually unknown. The aim of this study was to determine the complete genome sequence of *S. pluranimalium* strain TH11417 isolated from a cattle with mastitis, and to characterize its antimicrobial resistance, virulence, and carbon catabolism.

**Results:**

The genome of *S. pluranimalium* TH11417, determined by single-molecule real-time (SMRT) sequencing, consists of 2,065,522 base pair (bp) with a G + C content of 38.65%, 2,007 predicted coding sequence (CDS), 58 transfer RNA (tRNA) genes and five ribosome RNA (rRNA) operons. It contains a novel IS*Spl1* element (a memeber of the IS*3* family) and a Ф11417.1 prophage that carries the *mef*(A), *msr*(D) and *lnu*(C) genes. Consistently, our antimicrobial susceptibility test confirmed that *S. pluranimalium* TH11417 was resistant to erythromycin and lincomycin. However, this strain did not show virulence in murine pneumonia (intranasal inoculation, 10^7^ colony forming unit – CFU) and sepsis (intraperitoneal inoculation, 10^7^ CFU) models. Additionally, this strain is able to grow with glucose, lactose or galactose as the sole carbon source, and possesses a lactose-specific phosphoenolpyruvate-dependent phosphotransferase system (PTS).

**Conclusions:**

We reported the first whole genome sequence of *S. pluranimalium* isolated from a cattle with mastitis. It harbors a prophage carrying the *mef*(A), *msr*(D) and *lnu*(C) genes, and is avirulent in the murine infection model.

## Background

*Streptococcus pluranimalium* was first described as a new species of the *Streptococcus* genus in 1999 by Devriese et al. [1]. In sharp contrast with rather strict host restriction of many other streptococcal species, *S. pluranimalium* is promiscuous, in terms of its host and tissue tropism since it has been isolated from various tissues of multiple domestic animals and humans. In recent years, *S. pluranimalium* has been regarded as a pathogen associated with subclinical mastitis in dairy cows [1], many bovine reproductive diseases (abortion, stillbirth, vulvitis, vaginitis and metritis) [2], valvular endocarditis and septicaemia in adult broiler parents [3], septicaemia in Nile tilapia [4]. Furthermore, this bacterium has been isolated from human patients with subdural empyema, infective endocarditis, and brain abscess [5–7]. However, the biological properties and pathogenic mechanisms of *S. pluranimalium* are virtually unknown at the present time.

The previous *S. pluranimalium* isolates are oftern characterized by protein mass spectrometry and 16S rRNA sequencing [1, 3, 8]. Phylogenetic relationship of this species with the other members of the *Streptococcus* genus has been established with the sequences of selected genes (e.g. 16S rRNA, *rpoB*, so*dA*, *tuf*, *rnpB*, *gyrB*, *dnaJ*, *recN*, and *greL*) [9, 10]. Characterization of genomic features of this new member of the *Streptococcus* genus contributes to better understand its resistance, virulence potential and phylogenetic relationship among *Streptococci*. However, the complete genome of *S. pluranimalium* has not been reported. The aim of this study was to sequence and analyze the whole genome of a *S. pluranimalium* isolated from a cattle with mastitis. This strain was further evaluated, in terms of its antimicrobial resistance, virulence and carbon catabolism.

## Methods

### Strain and culture conditions

*S. pluranimalium* strain TH11417 was isolated in 2015 from the milk of a cattle with mastitis in Henan province, China. The strain was cultured in Todd-Hewitt broth (Oxoid Ltd., London, UK) supplemented with 0.5% yeast extract (THY) and on tryptic soy agar (Oxoid) with 5% (*v*/v) sheep blood at 37 °C. The 16S rRNA classification was performed according to a standard procedure using primers: Pr1 5’-AGAGTTTGATCCTGGCTCAG-3′ and Pr2 5′- ACGGCTACCTTGTTACGACTT-3′.

### Genome sequencing and analysis

The genomic DNA was extracted using Bacterial DNA Kit (Omega Bio-tek, Norcross, GA), according to the manufacturer’s instructions. The genome sequencing of *S. pluranimalium* TH11417 was performed on a PacBio RSII single-molecule real-time (SMRT) sequencing instrument (Pacific Biosciences, Menlo Park, CA). The average sequencing coverage was approximately 317× across the genome. The reads were assembled de novo using the hierarchical genome assembly process (HGAP) with the default settings of the SMRT Analysis v2.3.0 software package (Pacific Biosciences). The genome was annotated through the NCBI prokaryotic annotation pipeline (https://ncbi.nlm.nih.gov/).

The possible genomic islands (GIs) from TH11417 genome were predicted using IslandViewer 4 (http://www.pathogenomics.sfu.ca/islandviewer/), and prophage components were predicted according to the PHAST (http://phast.wishartlab.com/). Genome maps of TH11417 was generated using Circos v0.64 software [11]. The comparative analysis of prophage and type VII secretion system (T7SS) was generated using EasyFig v2.2 software (http://mjsull.github.io/Easyfig/files.html).

### Phylogenetic analysis

Phylogenetic tree was constructed using core genome containing 352 single-copy core genes of 68 members in the genus *Streptococcus* (67 *Streptococci* from NCBI GenBank and one in this study). The single-copy core genes were determined using the program OrthoMCL version 2.0 as described previously [12, 13]. The orthologous protein sequences were aligned and concatenated using ClustalW version 2.0 [14]. The concatenated proteins to infer the organismal phylogeny were analyzed using approximately-maximum-likelihood algorithm in FastTree version 2.0 [15]. The mapping of *S. pluranimalium* was generated by iTOL v4.0.3 (http://itol.embl.de/).

### Antimicrobial susceptibility testing

The antibiotic susceptibility was determined as minimal inhibitory concentration (MIC) using the broth microdilution method, following the guidelines of the Clinical and Laboratory Standards Institute [16]. The following antimicrobial agents were used: penicillin, cefotaxime, erythromycin, lincomycin, clindamycin, doxycycline, which were obtained from Sigma (Shanghai, China). *S. pneumoniae* ATCC 49619 was used as the quality control strain.

### Evaluation of the virulence of *S. pluranimalium* TH11417

The virulence of *S. pluranimalium* TH11417 was evaluated in murine pneumonia and sepsis models. Briefly, bacteria were grown to the mid-log phase and stored in 15% glycerol at − 80 °C for 2 days. Stocked bacteria were diluted in Ringer’s solution (RS) to appropriate dose for infection. For pneumonia model, groups of 6 female C57BL/6 mice (6–8 weeks old, Vital River, Beijing, China) were anesthetized by avertin through intraperitoneal (i.p.) injection and inoculated with 1 × 10^7^ CFU bacteria in 30 μL RS by intratracheal (i.t.) instillation. For sepsis model, mice were infected by i.p. with 1 × 10^7^ CFU bacteria in 200 μL RS. Every 24 h post infection, blood samples (20 μL) were collected from suborbital vein and plated on TSA plates with 5% (*v*/v) sheep blood for counting bacterial number, and the survival of mice were observed up to 7 days.

### Metabolism of carbohydrates

The metabolic capacity for carbohydrates was evaluated by monitoring the growth of *S. pluranimalium* TH11417 in the presence of different sugars as the main carbon source. Briefly, bacterial cells were cultivated in a chemically defined medium (CDM) as previously described [17], supplemented with 0.5% different carbon sources (glucose, lactose and galactose), respectively. Carbohydrates were purchased from Sigma (Shanghai, China). The growth phenotype was monitored by a BioTek Synergy H1 microplate reader (BioTek, Winooski, VT, USA) at 37 °C with 200 μl in each well, with the optical density at 620 nm (OD_620_) of each sample recorded every 30 min up to 24 h.

## Results

**Fig. 1:**
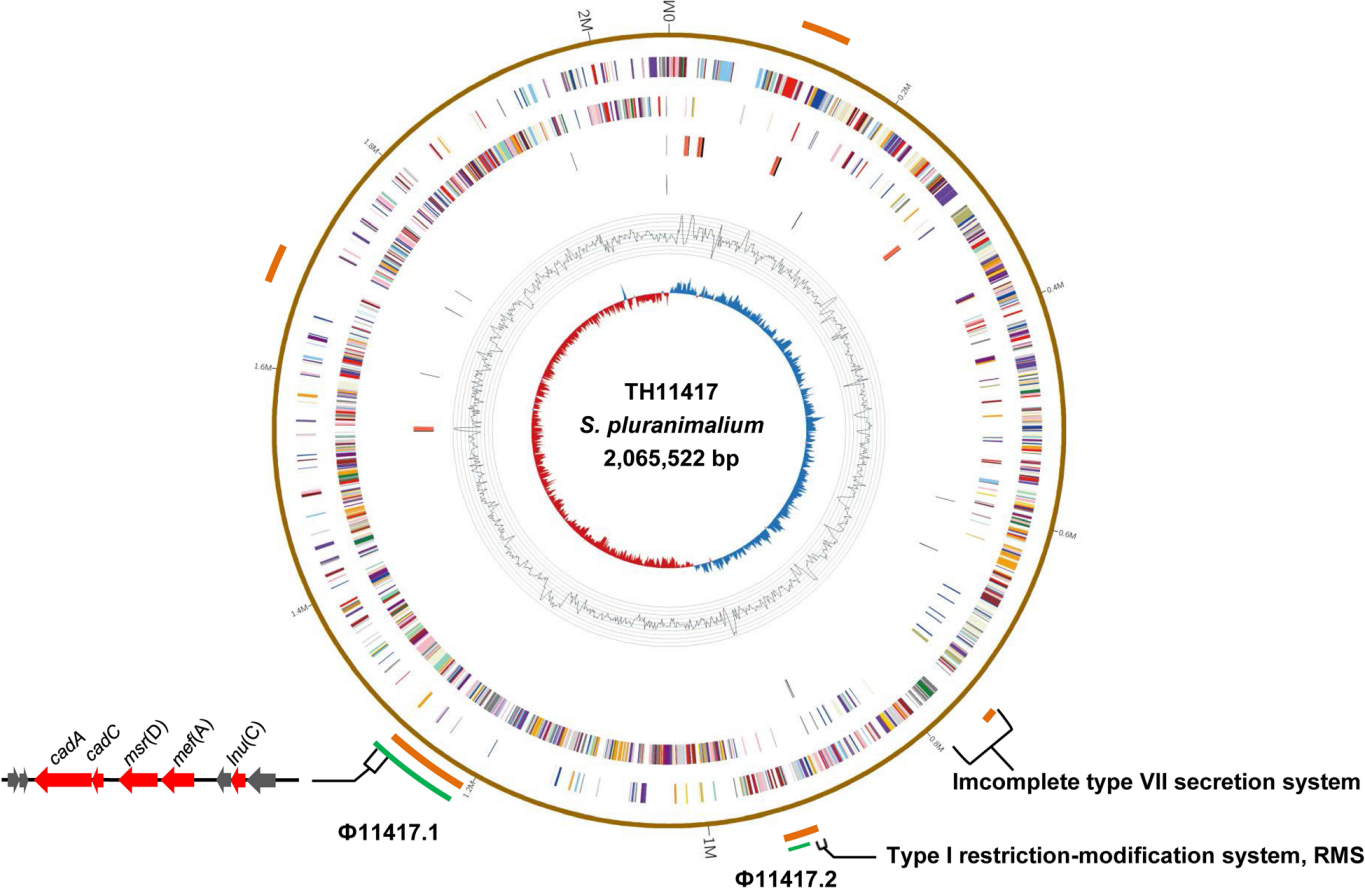
Genome map of *S. pluranimalium* TH11417. Map was established using the software Circos. The circular diagrams (rings from the outermost to the center): 1) prophages predicted (green), Ф11417.1 prophage carried the *mef*(A), *msr*(D) and *lnu*(C) genes; 2) genomic island (GI) (light red); 3) scale marks of the genome; 4) protein-coding genes on the forward stand; 5) protein-coding genes on the reverse strand; 6) tRNA (black) and rRNA genes on the forward strand; 7) tRNA (black) and rRNA genes on the reverse strand; 8) GC content; 9) GC skew. Protein-coding genes are color coded according to their Cluster of Orthologous Gene Categories (COG) categories [40]

**Fig. 2:**
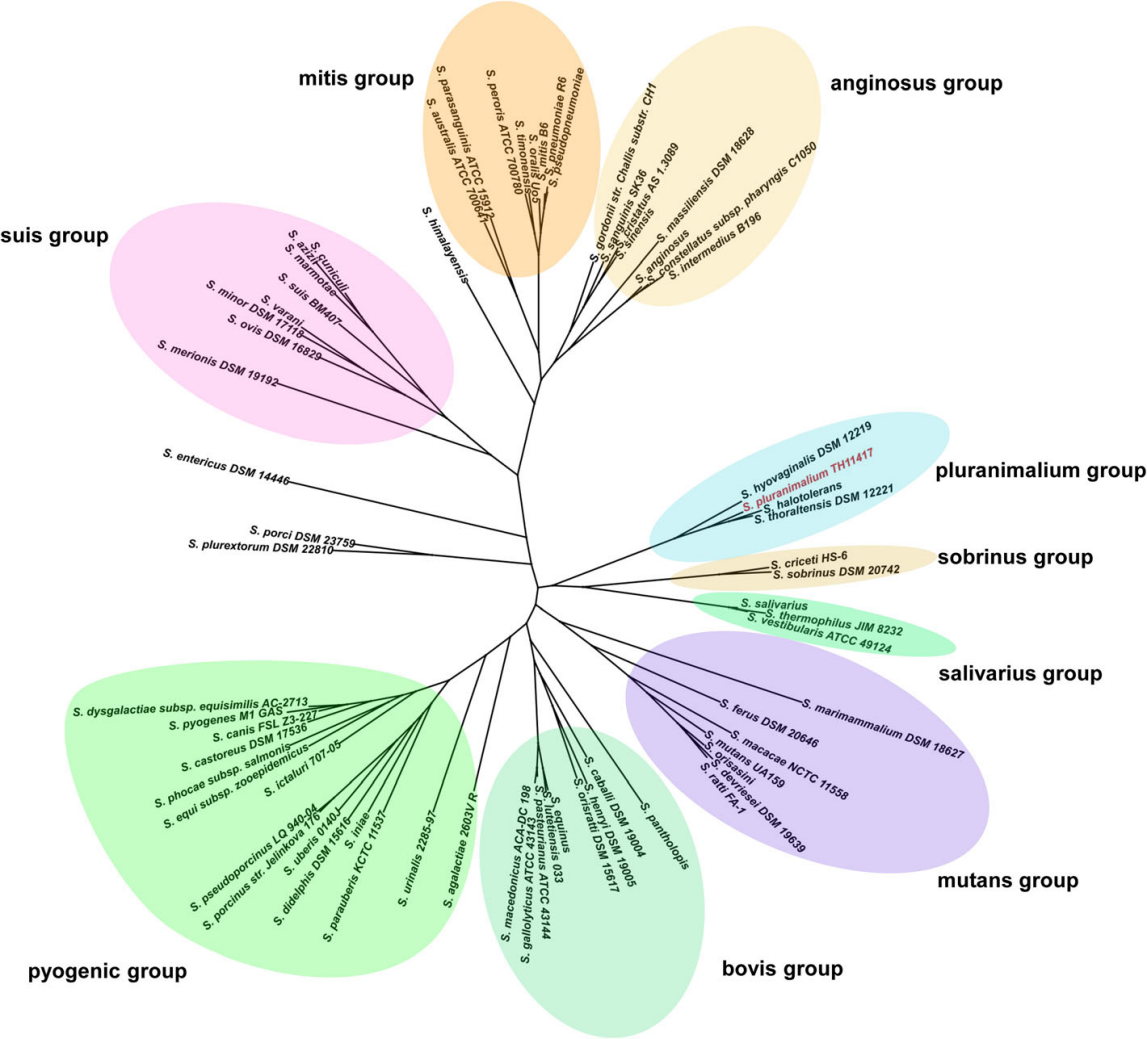
Phylogenetic tree of all 68 representative genomes in the genus *Streptococcus*, including *S. pluranimalium* TH11417. The tree was reconstructed with approximately-maximum-likelihood algorithm based on 352 single-copy core genes concatenated to core genome proteins, computed by FastTree and mapping by iTOL v4.0.3 (http://itol.embl.de/)

The TH11417 genome is composed of 2,065,522 bp with a G + C content of 38.65%. It consists of 2,007 predicted CDS, 58 tRNA genes and 5 rRNA operons (Fig. 1). Five genomic islands and two prophage regions were predicted by Island Viewer and PHAST, respectively. The first prophage, designed as Ф11417.1, is 52,668 bp in length and contains 53 CDS; the second prophage (named Ф11417.2) consists of 8,104 bp with 12 CDS. Phylogenetic analysis showed that *S. hyovaginalis*, *S. thoraltensis*, *S. halotolerans*, and *S. pluranimalium* form the *pluranimalium* group in the genus *Streptococcus* based on the distances calculated by approximately-maximum-likelihood algorithm (Fig. 2).

**Fig. 3:**
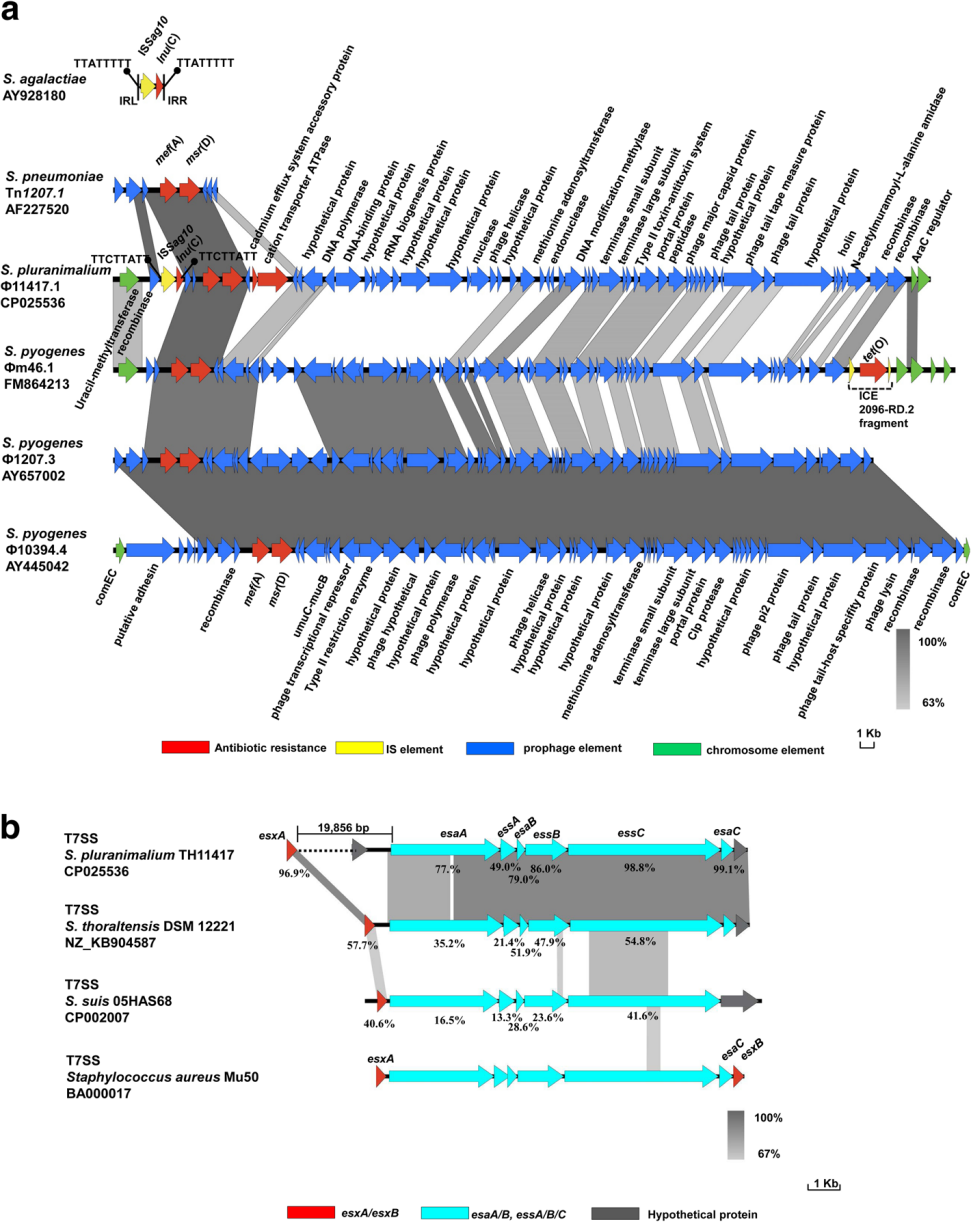
Comparative analysis of the Ф11417.1 and T7SS in *S. pluranimalium* TH11417 with that of other strains. **a** Sequence comparison between the Ф11417.1 in *S. pluranimalium* TH11417 with related genetic elements and prophages, IS*Sag10* bearing the *lnu*(C) gene in *S. agalactiae* (AY928180), Tn*1207.1* in *S. pneumonia* (AF227520), Ф1207.3 in *S. pyogenes* (AY657002), Ф10394.4 in *S. pyogenes* (AY445042), Фm46.1 in *S. pyogenes* (FM864213). The positions and orientation of transcription for resistance and other genes on each mobile genetic elements are indicated by directional arrows. Filled circles on stalks are used to indicate DRs. Homologous segments generated by a BLASTn comparison (≥ 63% identity) are shown as grey boxes. **b** Comparison of the *S. pluranimalium* TH11417 ESAT-6 locus with the *S. thoraltensis* DSM 12221 (NZ_KB9045876), *S. suis* 05HAS68 (CP002007) and *Staphylococcus aureus* Mu50 (BA000017) locus. The positions and orientation of transcription for T7SS locus are indicated by directional arrows. Homologous segments generated by a BLASTn comparison (≥67% identity) are shown as grey boxes. The identity of amino acid sequences for each gene related to the secretory antigenic targart ESAT-6 is expressed as a percentage

TH11417 was resistant to erythromycin (MIC = 16 μg/mL), lincomycin (MIC = 64 μg/mL), and susceptible to penicillin (MIC < 0.125 μg/mL), cefotaxime (MIC < 0.125 μg/mL), clindamycin (MIC = 0.25 μg/mL), doxycycline (MIC = 0.25 μg/mL). The analysis of whole genome indicated that it contains the *mef*(A), *msr*(D) and *lnu*(C) genes, which confer resistance to erythromycin and lincomycin. These resistance determinants are associated with a 52.7-kb chimeric genetic element composed of a transposon inserted into the Ф11417.1 prophage. This transposon contains the heavy metal transporter ATPase and efflux system accessory genes, *mef*(A) and *msr*(D) resistant genes, and a mobile element IS*Sag10* carrying *lnu*(C) gene. The IS*Sag10* is inserted to upstream of *mef*(A), generating two direct repeats (DRs) (TTCTTATT) (Fig. 3a).

A new 1,430-bp insertion sequence (IS) belonging to IS*3* family was identified in TH11417 and designated as IS*Spl1*. It is flanked by 20/25-bp imperfect inverted repeats and contains two open reading frames, which encode 178- and 304-amion-acid proteins. The whole IS*Spl1* shows 81% identity to the IS*861*, which was firstly characterized in *S. agalactiae* COH-I [18]. Four copies of IS*Spl*1 were observed throughout the chromosome of TH11417, and one of the copies lacking the target sequence is located near to the genes involved in bacteriocin synthesis, other copies create 3-bp directly repeated sequences at the target site (TTC, ATT, GGG) (http://www-is.biotoul.fr/).

Analysis of the whole genome of the TH11417 revealed that it harbors several virulence-associated factors, including fibronectin-binding protein, hemolysin III homolog, cell wall anchored protein sortase and LPXTG-anchored protein, IgA1 protease. The fibronectin-binding protein and hemolysin III protein of TH11417 display high identity at protein level with the same pluranimalium group and other streptococcal species whose genomes have been published in the NCBI database, 93.3 and 92.6% with *S. thoraltensis* DSM 12221 (NZ_KB904587), 91.4.0 and 88.9% with *S. halotolerans* (NZ_CP014835), 90.4 and 83.3% with *S. hyovaginalis* (NZ_ATVP01000004, NZ_ATVP01000012), respectively. BLASTp analysis showed that cell wall anchored protein sortase and LPXTG-anchored protein of TH11417 display moderate identity with that of many other streptococcal species. The IgA1 protease of TH11417 display low identity with that of many other streptococcal species. Together, the IgA1 protease in TH11417 has relatively higher specificity than other virulence-associated factors. As shown in Fig. 3b, the TH11417 genome carries a type VII secretion system (T7SS) harboring secretory antigenic target ESAT-6 (substrate protein, EsxA), secretion accessory protein EsaA and EsaB, secretion system component EssA, EssB, and EssC proteins. However, the T7SS locus is interrupted by many hypothetical genes between *esxA* and *esaA*. EsxA of TH11417 was found to show 96.9% amino acid identity to the corresponding protein of *S. thoraltensis* DSM 12221 (NZ_KB904587), moderate identity to that of *S. suis* 05HAS68 (CP002007) (58.8%) [19], and 44.3% identity to that of *Staphylococcus aureus* Mu50 (BA000017) [20]. The other related secretion proteins were illustrated in detail (Fig. 3b). In addition, the several hypothetical proteins of T7SS were predicted as genomic island by Island Viewer software. So, we speculated that this T7SS is incomplete and defective.

To verify whether these putative virulence factors confer pathogenicity to *S. pluranimalium*, TH11417 was used to infect mice at a dose of 1 × 10^7^ CFU in both acute pneumonia and sepsis models that have been used to assess the virulence of *S. pneumoniae* [21]. No bacteria were detected in the blood of mice infected by either i.t. (pneumonia) or i.p. (sepsis) 24 to 48 h post infection. All of the mice survived without any obvious symptom more than 7 days post infection. This result strongly suggested that TH11417 is relatively low- or avirulence.

**Fig. 4:**
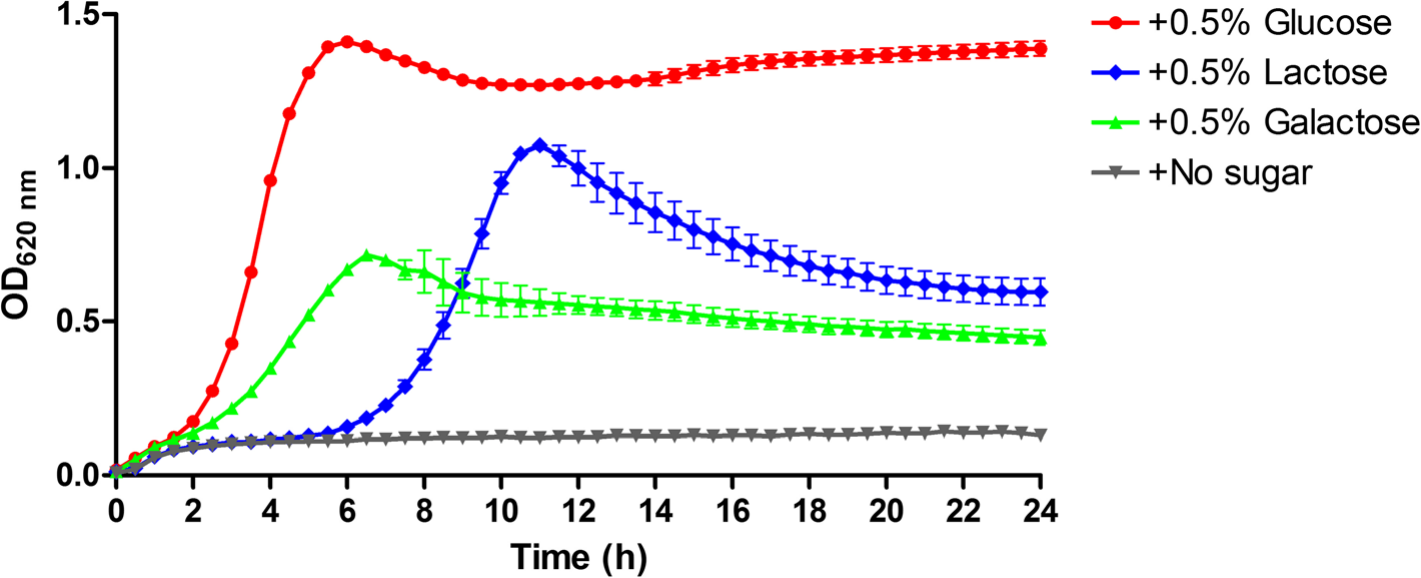
Growth curves of the *S. pluranimalium* generated using CDM medium supplemented with 0.5% glucose, 0.5% lactose, 0.5% galactose. The values are the means±standard deviations

**Fig. 5:**
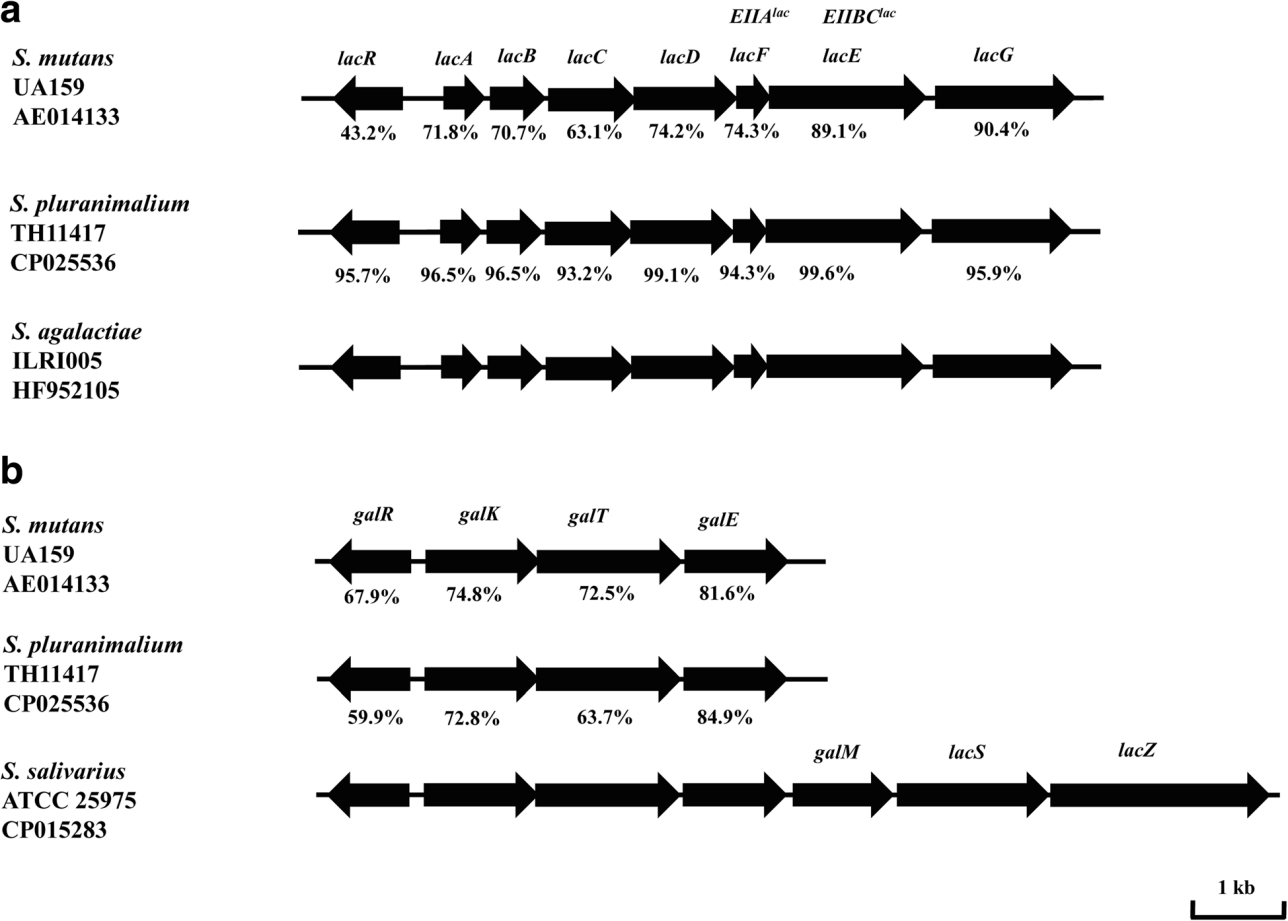
Organization of the galactose and lactose genes in the *S. pluranimium* TH11417. **a** Comparison of structures and indentity of *S. pluranimium* (CP025536), and *S. mutans* (AE014133) and *S. agalactiae* (HF952105) *lac* locus genes. The directions of genes are indicated by arrows. Percent identities of amino-acid sequences are indicated. **b** Comparison of structures and indentity of *S. pluranimium* (CP025536), and *S. mutans* (AE014133) and *S. salivarius* (CP015283) *gal* locus genes. The genes code for the following proteins: *lacR*, the DeoR-like negative transcriptional regulator; *lacA* and *lacB*, the A and B subunits of the galactose-6-phosphate isomerase; *lacC*, the tagatose-6-phosphate kinase; *lacD*, tagatose-1,6-bisphosphate aldolase; *lacF* and *lacE*, the A and BC components of the lactose-PTS enzyme II; and *lacG*, the 6-phospho-β-galactosidase; *lacS*, lactose permease; *lacZ*, β-galactosidase; *galR*, putative transcriptional regulator; *galK*, galactokinase; *galT*, galactose-1-phosphate uridylyltransferase; *galE*, UDP-glucose 4-epimerase; *galM*, galactose mutarotase

The ability of *S. pluranimalium* TH11417 to grow with glucose, lactose and galactose as the main carbohydrate source was evaluated in CDM medium supplemented with single carbohydrate. As presented in Fig. 4, TH11417 grew in the presence of glucose, lactose, or galactose. As expected, the CDM with glucose yielded the most productive growth as evidenced by the doubling time in the exponential phase (6 h) and maximal culture density (OD620 1.4). In contrast, the medium containing lactose or galactose showed much slower growth. Although TH11417 showed the longest lag phase in the lactose CDM but eventually showed a second highest maximal density (OD620 1.0), suggesting that lactose metabolism requires extra time for induction. Analysis of the TH11417 genome revealed that it harbors intact lactose and galactose metabolism loci (*lacRABCDFEG* and *galRKTE*). The lactose metabolism locus consists of 8 genes (*lacR* and lac operon of 7 genes: *lacABCDFEG*). The genes in the *lac* locus of *S. pluranimalium* are highly similar to those of *S. agalactiae* ILRI005 in gene organization and amino acid sequence [22] (Fig. 5a). As an example, *lacC*, the least similar gene in the locus between the two species, has 93.2% sequence identity. In contrast, the *lac* operon of *S. pluranimalium* has much lower overall sequence homology with that of *S. mutans* UA159 [23, 24], a well-characterized oral streptococcus (Fig. 5a). The *gal* operon (*galRKTE*) in TH11417 also has the same organization as in *S. salivarius* ATCC 25975 [25], however, the lactose permease *lacS* is absent (Fig. 5b). These results indicated that *S. pluranimalium* TH11417 is capable of transporting and metabolizing lactose though lactose PTS and tagatose 6-phosphate pathway.

## Discussion

*S. pluranimalium*, was first identified by Devriese et al. In 1999 [1]. Since then, this new *Streptococcus* was isolated from different animals and humans. However, the complete genome of *S. pluranimalium* is still unknown. In this study, we determined the complete genome sequence of *S. pluranimalium* TH11417. The genus *Strepococcus* has been divided into nine major groups (mutans, bovis, pyogenic, suis, mitis, anginosus, pluranimalium, sobrinus, and salivarius) [10]. Phylogenetic analysis of the TH11417 genome has confirmed that *S. pluranimalium* forms the *pluranimalium* group with *S. hyovaginalis*, *S. thoraltensis*, and *S. halotolerans* (Fig. 2). Moreover, the *S. pluranimalium* genome is closely related to the streptococcal genomes in the sobrinus and salivarius groups, suggesting that pluranimalium is ancestral to these two groups (Fig. 2). Notably, *S. gordonii* belongs to mitis group based on analysis of the 16S rRNA gene [26], whereas *S. gordonii* was classified as anginosus group by single-copy core genes as well as called gordonii group using eight phylogenetic markers [10].

This study, for the first time to our best knowledge, revealed that three drug-resistance determinants *mef*(A), *msr*(D) and *lnu*(C) coexist in a single prophage. The *mef*(A) gene encodes an efflux pump exhibiting resistance to macrolides, and susceptibility to lincosamides and streptogramin B antibiotics, which was originally described in *S. pyogenes* in 1996 [27]. The *msr*(D) gene, one of the ABC-F subfamily of ATP-binding cassette proteins, mediate macrolide resistance through ribosomal protection [28]. The *msr*(D) gene along with *mef*(A) was previously found on the defective transposon Tn*1207.1* in *S. pneumoniae,* which could not be transferred by conjugation experiment [29]. However, an originally called Tn*1207.3* conjugative transposon carrying this *mef*(A)/*msr*(D) pair of genes could be transferred in different streptococcal species. Now, the Tn*1207.3* was re-named as a prophage Ф1207.3 in *S. pyogenes* [30]. Including *lnu*(C) gene conferring resistance to lincomycin, several different genes have been identified and deposited in the nomenclature centre for MLS resistance genes (http://faculty.washington.edu/marilynr/), which inactivate lincosamides by adenylylation in *Staphylococcus, Enterococcus, Streptococcus, Haemophilus parasuis, Riemerella anatipestifer*. The IS*Sag10* bearing *lnu*(C) was first identified in *S. agalactiae* UCN36 in 2005, which was inserted in the operon for capsular synthesis, and generated both DRs (TTATTTTT) [31]. In the present study, the IS*Sag10* is simply inserted to a transposon resembling Tn1*207.1* of *S. pneumoniae* [29] (Fig. 3a). At the sequence level, Ф11417.1 has low homology to Фm46.1, Ф1207.3 and Ф10394.4 from *S. pyogenes*, except for Tn1*207.1*-like elements [30, 32, 33]. Interestingly, the Ф1207.3 and Ф10394.4 integrate into *comEC* coding sequence at the same chromosomal site, whereas Ф11417.1 as well as Фm46.1 integrates into the gene encoding 23S rRNA uracil methyltransferase (Fig. 3a). These results indicated that *S. pluranimalium* TH11417 could acquire the resistance determinants through phage horizontal transfer.

This study has identified a type VII secretion system (T7SS)-like locus in *S. pluranimalium.* T7SS, the newest secretion system in prokaryotic organisms, are found in certain Gram-positive pathogens, including *Mycobacteria tuberculosis* and *Staphylococcus aureus* [34]. Very recently, Lai et al. reported a type VII secretion system in *S. suis* which contributes to virulence in a mouse infection model [35]. Although multiple virulence associated factors are found in the genome of *S. pluranimalium* TH11417, this strain did not show obvious virulence in both the pneumonia and sepsis mouse models. Because previous studies have shown that *S. pluranimalium* is associated with diseases in domestic animals and humans [1–6], it is possible that TH11417 is specialized in colonizing the bovine environment and lacks certain factors for successful infection in mice. The availability of the TH11417 genome will help future investigations into the genetic basis of pathogenesis and biology in this species.

Lactose is the primary carbon and energy source used by some *Streptococcus* strains for growth in milk [36]. In this study, we isolated *S. pluranimalium* TH11417 from a cattle with mastitis, which is capable of metabolizing lactose and galactose. There are multiple systems to transport/metablize a single substrate in bacteria [36, 37]. In lactose metabolism, the β-galactosidase (LacZ) is the predominant metabolic system through lactose permease (LacS) for *S. salivarius* 25975, while the lactose-PTS is the major metabolic pathway for *S. mutans*, both of which were induced by lactose [37]. Like bovine-adapted *S. agalactiae* [38], *S. pluranimalium* TH11417 could also metabolize lactose and galactose by two distinct pathways: tagatose 6-phosphate (*lac*) and Leloir (*gal*) passways. In *S. salivarius* and *S. thermophilus*, lactose is not transported by lactose-specific PTS, but solely through lactose permease (LacS), which is cleaved by β-galactosidase (LacZ). However, *S. salivarius* is able to metabolize galactose via the Leloir pathway, while *S. thermophilus* doesn’t metabolize galactose because *galK* gene is poorly translated [25, 39]. In the present study, TH11417 strain harbors intact *lac* and *gal* operons, but the lactose permease *lacS* is absent. The genotypes are consistent with the poor growth in CDM medium with 0.5% lactose during the first 6 h of incubation, suggesting that the lactose-PTS is the primary metabolic pathway for lactose.

## Conclusions

In conclusion, we reported the first whole genome sequence of *S. pluranimalium* isolated from a cattle with mastitis. The analysis of whole genome revealed that TH11417 harbors a chimeric Ф11417.1 prophage carrying Tn*1207.1*-like and IS*Sag10* transposons, and several putative virulence factors, such as a fibronectin-binding protein and a type VII secretion system-like locus. *S. pluranimalium* TH11417 transports and metabolizes lactose though lactose PTS and tagatose 6-phosphate pathway. This complete genome will be highly valuable for the genetic basis of biology and pathogenesis in this species.

## Abbreviations

CDM: Chemically defined medium
CFU: Colony forming unit
IS: Insertion sequence
MIC: Minimal inhibitory concentration
T7SS: Type VII secretion system
